# Constructivist multi-criteria model to support the management of occupational accident risks in civil construction industry

**DOI:** 10.1371/journal.pone.0270529

**Published:** 2022-06-28

**Authors:** Leonardo Ensslin, Alex Gonçalves, Sandra Rolim Ensslin, Ademar Dutra, André Andrade Longaray

**Affiliations:** 1 Department of Administration, University of Southern Santa Catarina, Florianópolis, Santa Catarina, Brazil; 2 Department of Accounting, Federal University of Santa Catarina, Florianópolis, Santa Catarina, Brazil; 3 Department of Economic, Administrative and Accounting Sciences, Federal University of Rio Grande, Rio Grande do Sul, Brazil; SASTRA Deemed University, INDIA

## Abstract

Civil construction is one of the industrial sectors with continuous growth globally, particularly in Brazil in the last 50 years. Unfortunately, it is also one of the productive segments with the highest incidence of accidents, which overshadows its merits as a driver of economic growth and job creation. The damage to workers’ health caused by accidents at work results from the presence of work environment risk factors. Therefore, this study aims to manage these risk factors for the civil construction industry. The work is original with respect to building a model to support risk management in civil construction for a specific and relevant context. It is ensured by presenting an unprecedented approach to the sector that incorporates information not considered by classic generic approaches. This research, thereby, seeks to build a model to support the risk management of accidents in the workplace in the prefabricated concrete construction industry. It is a case study with a constructivist approach and an exploratory and descriptive character, incorporating the Multicriteria Methodology for Decision Aiding-Constructivist (MCDA-C). The main findings include (i) identifying the strategic objectives: occupational safety policy, work environment, machines and equipment, condition of materials, procedures and methods, and skills, which were operationalized via 58 criteria; (ii) examining the scales of the criteria such as the performance profile of the current situation and the goal, highlighting the vulnerabilities and potentials; (iii) proposing improvement actions for the vulnerabilities, thus supporting risk management in the organization. Among the contributions, managers and professionals in the field contribute to the possibility of using an instrument customized to the context and legitimate to their concerns and values stands out. Furthermore, the contributions of researchers include the challenge of improving their generic models with the knowledge of personalized models.

## 1. Introduction

Civil construction has a diversity of products and areas of operation: roads, bridges, viaducts, tunnels, subways, telecommunication towers, houses, buildings, among others, and more recently, prefabricated industries. All of them carry the stigma of being fatal or are activities that engender disabling accidents, because most of them use low-skilled labor resulting in painful social and economic consequences. Even in developed countries, the civil construction sector yields other productive sectors, all of them leading to several occupational accidents [1–3].

Being a sector that traditionally demands low resources in fixed assets, many investors focus their attention on this segment in moments of reduced business opportunities. The disorderly increase in the construction of buildings in recent decades has led to unbridled competition among companies, instigating them to rethink their business and seek innovative ways to compete.

In this context, one finds the concrete prefabricated construction industry, which seeks to combine products of excellence with work quality benchmarks, equated to the best international ones [4, 5]. The methods used in the development of prefabricated concrete construction, despite being largely repetitive processes for historical reasons, incorporated technological advances in their equipment. However, the processes and layout did not keep pace with engineering developments in terms of occupational safety and health [6, 7].

Although prefabricated construction can be considered a sustainable method that leads to market growth [8] and is safer than traditional construction, accidents still occur [9]. Therefore, there is an opportunity to reduce accidents at work [10].

The existing construction safety literature includes only a few cases that address safety risks and hazards in the prefabricated building sector. However, Fard et al.’s [6] research targeted the prefab industry to discover practices that need to be addressed to improve safety performance. In their findings, the predominant causes of work accidents were: “fall,” “struck by object/equipment,” and “caught in equipment/object/material.” The same authors conclude that the prefabricated construction strategy can be challenging and risky in terms of safety. For example, when moving and installing massive components, when lifting, moving, and installing large and heavy components [6].

Li et al. [7] link the risk factors to the prefabricated construction process as (i) General risk factors: these are the common risk factors that affect both on-site and off-site construction, including economic conditions, political and social conditions, planning and construction; (ii) Factory risk factors: These are the factors involved in off-site prefabrication of modules and panels, material quality, workmanship skills and condition of fabrication equipment. (iii) Site risk factors: these are the factors impacting on-site preparation and installation, including site condition and construction equipment condition [7].

Recent surveys by the International Labour Organization (ILO) report that about 2.78 million workers lose their lives each year globally due to accidents at work or occupational diseases, and 374 million workers are victims of non-fatal accidents at the workplace [11]. In 2020, Brazil alone registered 1866 deaths because of 446.9 thousand occupational accidents [12]. Estimates at a global level say that lost working days account for almost 4% of world GDP [13]. In addition to the economic loss, there is also an intangible cost (not reflected in these numbers) that is represented by the psychic effects resulting from accidents, which atrophy their productive potential in addition to the immeasurable human suffering caused by occupational accidents. This reality is dramatic, as this suffering is avoidable to a large extent.

Damage to workers’ health caused by accidents at work notably results from the presence of risk factors in the work environment. The risks endanger the workforce, equipment, the working environment, and the enterprise itself [14]. In one of the classic studies on the causes of accidents at work, Heinrich introduced the domino theory [15]. According to Heinrich, the occurrence of accidents or injuries can be explained by five dominoes that represent (i) ancestry and social environment, (ii) fault of a person, (iii) unsafe act and mechanical or physical hazards, (iv)) accident and (v) injury. These dominoes fall and create a chain of events that results in an accident causing injury to workers [15].

Considering this, organizations often adopt safety management system approaches to manage the risks of accidents at work to eliminate or, at least control these undesirable events [7]. From 1980 onwards, the first models of risk management systems in the construction industry were designed [16]. These models successfully describe accident causality with comprehensive factors and allow support for investigation methods. However, risk management methods currently predominate with a limited number of measurable factors in their analysis [17].

This is reflected in several international surveys that have been conducted in the last decade [1–3, 18–22]. In 2015, Podgórski suggested that new approaches be researched and implemented to improve the effectiveness of occupational accident risk management models [20].

This evolving environment marked by the search for new forms, procedures, technologies, and a vision of risk management, while alerting to the need for new approaches, expands and makes security levels more demanding. The already complicated environment in which the context that demands risk management is located has become more complex. It includes multiple actors with different objectives, asymmetry of power, and greater demands on the part of workers and especially on society and bodies of oversight. These factors need to be considered in risk management support models [23].

The works developed in this area [1–3, 18–22] focus their efforts on researching the universal factors that generate risk in civil construction, which has been shown to contribute. However, this view ignores relevant knowledge-generating factors resulting from non-observance of the (i) singularities of the context; (ii) concerns and values of those responsible for management; (iii) use of measurement scales to inform the degree of vulnerability of each factor; (iv) the integration of factors to know global vulnerability; (v) the use of global knowledge to generate measured improvement actions and therefore liable to be ordered according to their contribution. These gaps were the motivation for the present study to determine approaches that incorporate these gaps in their processes.

Dealing with contexts that involve aspects of risk management, particularly in prefabricated concrete construction works, according to Rittel and Webber [24], Roy [25], and Landry [26], demands the use of soft approaches that focus their efforts on the construction of knowledge. Subsequently, it clarifies how the consequences of risks affect the strategic objectives of managers and the organization. The area of knowledge that has shown success in dealing with these contexts is multi-criteria performance assessment in decision support [27–31].

Multicriteria approaches in decision support when developing models to expand knowledge and identify, organize and measure the performance of factors considered essential in a unique way to the physical environment, and the actors involved aligns with the purposes of risk management in a way that meets scientific requirements and user demands [32, 33].

This vision benefits risk management by highlighting the properties of the context that are potentially more susceptible to accidents and the levels of risk associated with it. Early knowledge and dissemination of these events allow us to act proactively.

Considering the exposure, a research question emerges: Which criterion should be considered when assessing the management of occupational accident risks in the concrete prefabricated industry? The present study attempts to respond to this query through its general objective: to build a constructivist multicriteria model to support the management of occupational accident risks in the concrete prefabricated construction industry, from the perspective of a decision maker. The specific goals are: (i) to identify the decision-making context and the actors for which the model was built; (ii) to determine the strategic objectives and structure a set of criteria consisting of ordinal and cardinal scales, which makes it possible to highlight the performance profile of the current situation and the goal, highlighting the vulnerabilities and potentialities; and (iii) to propose improvement actions aimed at mitigating the risks of accidents at work in the organization.

Given the complexity of the situation, with a high number of objectives with conflicting purposes, the Multicriteria Methodology for Decision-Aiding Constructivist (MCDA-C) was selected as the intervention instrument to support the model-building process.

The importance of this study is evaluated from the perspective of originality and relevance. Although there is scientific research that proposes different methods of risk management using a realistic approach [1–3, 18–22], there are no available studies that employ the constructivist multi-criteria model to support the management of occupational accident risks. Hence, this study is unique as well as relevant as it directly relates to the social issue of occupational health, and approaches it from a novel perspective.

## 2. Materials and methods

### 2.1. Methodological framework

The philosophical concept adopted in this research was constructivism, which assumes that the subject (environment) and the actors integrate in a unified manner to form a unique entity. It is assumed that one wishes to model this entity in order to support its management and that the model provides the decision maker the knowledge about how the properties of the context affect their values [25, 34]. According to Ensslin et al. [27] from a constructivist perspective, the objective of modeling is to generate knowledge for decision makers from their values, preferences, and concerns in the decision-making context.

### 2.2. Intervention instrument for structuring the multicriteria model for decision-aiding constructivist (MCDA-C)

The intervention instrument applied to the study was Multicriteria Methodology for Decision Aiding-Constructivist (MCDA-C). According to Ensslin et al. [35], the method has had its scientific rise since the 1980s, with the publications of Roy [25], Landry [26], Rittel and Webber [24], and Bana e Costa [34].

The MCDA-C emerges as an alternative to the traditional MCDA to support decision-makers in complex, conflicting, and uncertain contexts [36, 37]. Considering the theoretical and methodological terms, classical MCDA restricts decision support to a stage of definition of objectives with little or no participation of the decision-maker, usually from a set of previously defined alternatives [38, 39]. However, the MCDA-C methodology has its main vocation. It is the process of expanding the decision maker’s knowledge about the context, culminating in the generation of an aggregation model to a single synthesis criterion formed by all the criteria perceived by the decision-maker as essences to the context management process. This occurs in a systemic and systematic way [36, 37].

The MCDA-C has been used in the last decade as an alternative for building models in different decision-making contexts. Rodrigues et al. [40] conclude that using the method can improve the view on the assessment of organizational climate and contribute to the decision-making process in health institutions. Corrêa Chaves et al. [41] use MCDA-C to support the development of management system assessment software. To support organizational knowledge management in the public sector, Ensslin et al. [27] adopted the constructivist model. Longaray et al. [42] evaluate the management process of a fertilizer industry with the support of the MCDA-C. Ensslin et al. [43] developed the model in the banking sector. The topic of solid waste management in small towns was addressed by Rodrigues et al. [44] with the support of the methodology. To present a decision-making methodology used to support R&D management in a technology-based company, Marafon et al. [45] applied the method to manage innovation. Della Bruna Jr. et al. [46] use the MCDA-C to evaluate the supply chain operations of an organization in the refrigeration equipment sector. Using the MCDA-C, Azevedo et al. [33] developed a framework to evaluate budget performance for apartment building projects.

**Fig 1:**
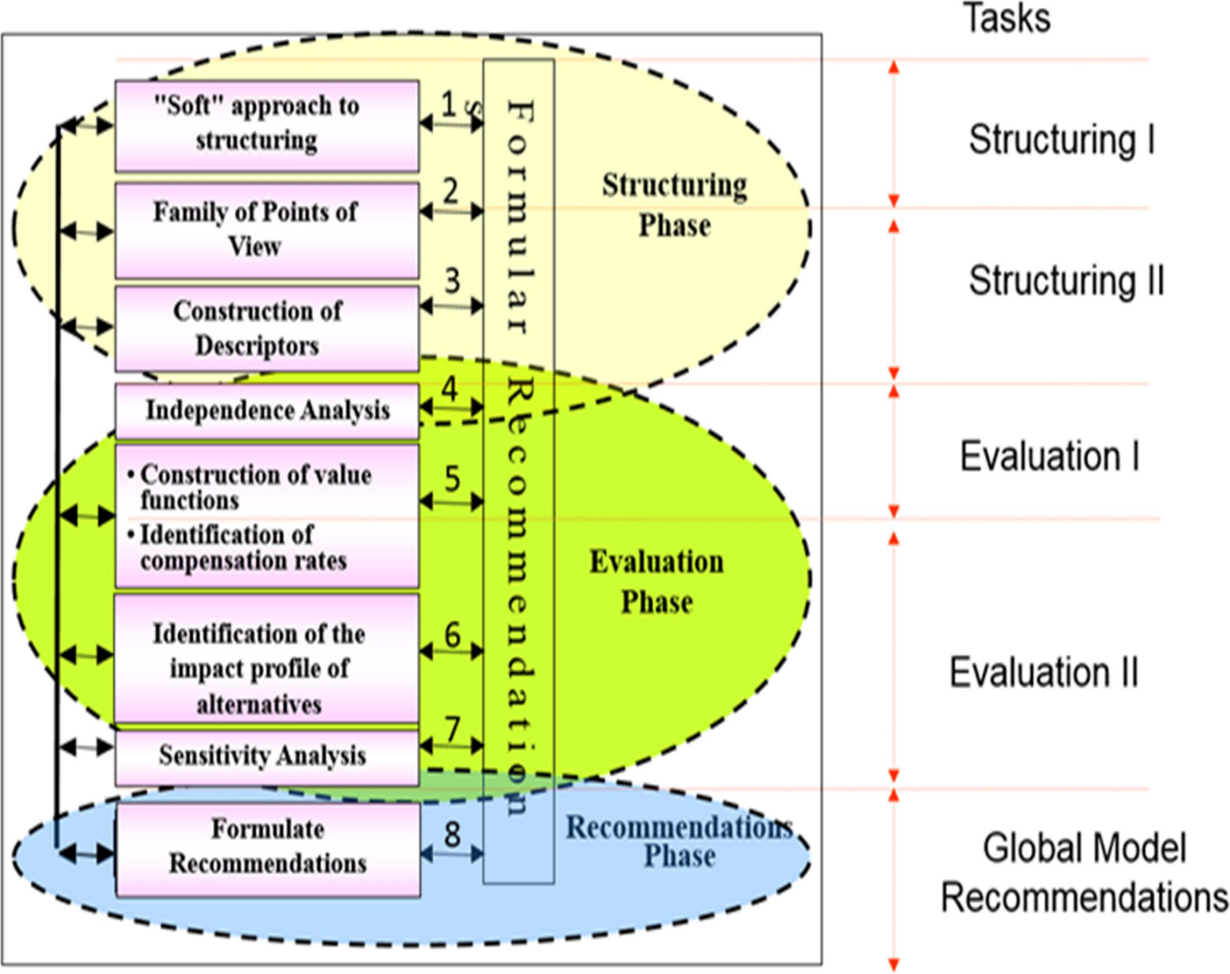
Operationalization of MCDA-C and its phases.

Ensslin et al. [35] represent the operationalization of the MCDA-C methodology through three basic, differentiated but intrinsically correlated, phases: (i) structuring the decision-making context; (ii) building a model for evaluating alternatives and actions; and (iii) formulating recommendations for the most satisfactory courses of action, as represented in Fig 1.

The constructivist model consists of building a cluster of tools in an interactive way with the decision-making stakeholders that allows progress in the structuring process in a manner consistent with the objectives and values of the decision-makers [25].

**Fig 2:**
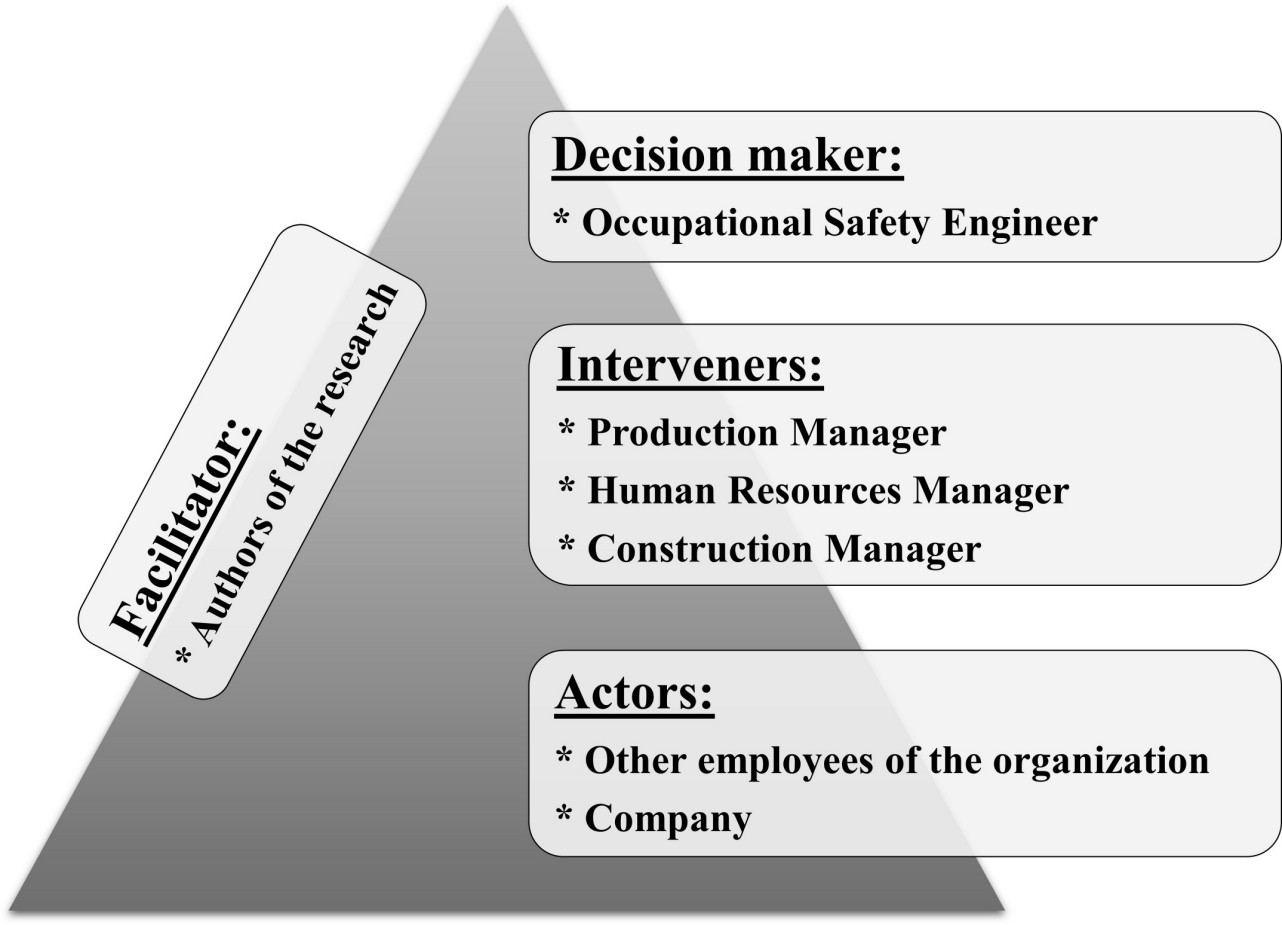
Identification of actors in the context.

The first phase (i) of structuring the decision-making context is “soft approach structuring,” which consists of identifying the actors (Fig 2) and interviewing them to specify their personal values, motivations and beliefs, the rules and context limits, and its characteristics that define the decision-making context. This phase is the most critical in terms of the decision support process, as it is here that the problem and the context in its breadth and boundaries will be defined, also generates a description of the organization and allows the decision makers to develop and expand their knowledge of the business context [39, 47, 48].

The next stage is called “family of points of view,” which is also done by open interviews with the actors speaking freely about the context for which they wish to identify the aspects essential to their management such as concerns, desirable characteristics, potential actions, objectives, restrictions and recurring problems [35, 47].

In this section of the structuring phase, the most critical factors of success (or failure) of the company’s competitive capacity, for the context under study, were identified. Each key factor—that we will call Fundamental Point of View (FPV)—was then made operational, for the evaluation of the characteristics considered relevant in the context, through the construction of a descriptor (ordinal scale) of the plausible impact levels in that FPV.

Based on this understanding, the facilitator proposes a scale that meets the principles of objectivity, precision, and accuracy [25] and represents the understanding of decision makers about what he considers important to be measured, and defines good and neutral reference level thresholds. The upper reference level (good), indicates performance judged to be at the level of excellence, while the lower level, (neutral), indicates the threshold below which performance is deemed to be compromising. Between these two thresholds, the performance is considered competitive or at the level of normality [27, 28].

The introduction of reference levels is determinant to allow an absolute assessment with the descriptor and to allow the analysis of the preferential independence (isolability) and integration of the criteria [28, 38] The importance of FPVs becomes clear in the hierarchical value structure, (Fig 7). This analysis, in turn, enabled the design of a profile of impacts for the dimensions in which, the firm had better competitive skills and those where it was more vulnerable.

The second phase (ii) building a model for evaluating alternatives and actions aims at building an alternative evaluation cardinal model. In this phase, the ordinal scales are transformed into cardinal (interval) scales and the criteria are integrated into a simple additive aggregation model via the following steps: the ordinal and cardinal preferential independence test of the scales for the interval between the reference levels is conducted; the ordinal scales are transformed into cardinal scales; the compensation rates or substitution rates are constructed; the impact profile of alternatives is identified; and sensitivity analysis is performed [27, 36, 47].

The purpose is to transform the descriptors scales developed in the structuring phase into interval scales and determine the compensation rates to integrate the criteria [36, 42, 49]. The decision-makers preferred value judgments regarding the difference in attractiveness between the levels of the scales were used to construct the value functions (interval scales) and regarding the difference in attractiveness between the reference levels of each scale to determine the compensation rates [28, 50]. The model provided information to the decision maker for the later creation of strategic intervention actions (see section 3.3).

It is important to remark that the construction of the model was a recursive process. Such an approach made MCDA more versatile and flexible since it allowed feedback in and to any stage. Acting in this way enabled the actors to progress in their learning process without anxiety, for they could go back at any time, if desired, and make it possible to compare two actions in terms of preference and to support the choice, even with conflicting aspects found among different actors.

The third phase (iii) formulating recommendations for the most satisfactory courses of action, the researchers establish a process to understand the advantages and disadvantages, and to create improvement actions in the decision-making context [28]. The possibility of improving the impact of the performance in an FPV, from its actual level to the reference impact level “good,” provided a strategic opportunity for potential action in the firm. Of course, the marginal increment on the overall value of the company depends not only on “how far” its impact in that FPV is from being good, but also on how significant the respective weight is.

This phase, according to the MCDA-C protocol, seeks to develop strategies (sets of actions) that enable current performance to be analyzed to identify actions that allow the goal to be achieved or even exceeded [27]. The recommendation phase generates proper understanding for the decision makers, so that they can comply with performance criteria judged essential and comprehensive for the given context. Thus, this phase shows strengths (excellent performance level) and issues with poor performance (Fig 7).

This knowledge allows the decision-makers to improve performance in those areas that they consider the most important. The process for the identification of potential actions for improving the impacts of the company has been harder for some FPVs than for other ones. Once the criterion that provided the greatest contribution was identified, the facilitator identified the property that has its performance measured by the criterion and requested the decision maker’s help in identifying actions to move the performance in this property from current performance to target performance [35]. Fig 8 shows the improvement actions generated for the criterion “Near accidents”, measured by the decision maker. The entire process was carried out following the MCDA-C protocol as shown in Fig 1. The operationalization of the three phases of the MCDA-C is described in the following section.

## 3. Results

### 3.1. Model structure

Structuring is the initial phase of MCDA-C, and it aims to understand the problem and the context in which it is inserted [28]. This stage contributes to the identification, organization, and ordinary measurement, which reflects the concerns of the decision maker for the decision-making situation uniquely and legitimately [25, 26].

#### 3.1.1. Contextualization, actor subsystems, and labeling

Contextualization is a stage in the process of modeling decision-making environments where the actors and their functions are reported as well as the physical environment where the problem is inserted. The context involves a situation perceived by the actors as complex, with a high number of objectives with conflicting, partially defined purposes, where the decision maker wishes to expand their understanding of how their decisions affect their values and motivations. The model is aligned with their perception of the context.

Given that it is a constructivist methodology, the process begins with the identification of the actors. Next, these same actors present the context highlighting their delimitations and environments contemplated [47]. Fig 2 presents the actor subsystems involved in the context.

The models built from the MCDA-C aim to represent the decision maker’s perceptions. Therefore, he is the most important component of the model construction process. The other stakeholders (actors) participate because they are part of the environment, and thus, being in the interest of the decision-maker, having them committed to the management support instrument and recognizing the model as theirs is therefore legitimate. Since understanding does not pre-exist and must be built, the process is iterative and evolutionary. Consequently, it causes the model elements to undergo adjustments until they reach their final form, in terms of essential factors such as their scales, reference levels, and degree of importance.

The decision-maker is the organization’s occupational safety engineer. Among his attributions, he must ensure that the task procedures and the current technical standards are compiled to promote the health and safety of the workers in the performance of their functions. The option of choosing the safety engineer as a decision-maker is due to the fact that he is responsible for the healthiness of the work environment and the knowledge of risk management of work accidents and their impacts on the organization. The identification of decision-makers as professionals who work directly in the management of safety at work was also found in the studies by Wachter and Yorio [51], Fung and Tam [18], Fernández-Muñiz et al. [52], Fung et al. [3] and Yiu et al. [16].

As interveners, who interact and are directly affected by the context, there are production, human resources, and construction managers. Production management is responsible for guaranteeing performance, besides ensuring production targets within the established standards of quality, quantity, costs, and deadlines. Human resources management, conversely, is responsible for managing people to develop talent within the organization, keeping the team cohesive and competent to meet the company’s demands. The works manager, is responsible for the final stage of the process, in which the constructions are carried out with the assembly of prefabricated concrete parts. The group of interveners was of great importance during the validation of each stage, as they were responsible for implementing the results (models) of this work.

Through discursive interaction with the actors, the facilitator encouraged the decision maker to talk openly about the context. After reflection, the most appropriate label for the research was defined, resulting in the “Construction of a Model to Support the Management of Occupational Accident Risks in the Prefabricated Concrete Construction Industry.” The physical environment was represented by industrial facilities producing prefabricated elements on demand for civil buildings.

#### 3.1.2. Primary assessment elements, concepts, and areas of concern

The MCDA-C methodology protocol guided the facilitator to establish a discursive interaction with the decision maker, and from this, the required data was extracted to build the model. At this stage, the decision maker was encouraged to speak openly about aspects of the problem, such as concerns, desirable characteristics, potential actions, objectives, constraints, and recurring themes. From this discussion, the facilitator extracted the data representative of contextual properties perceived as essential to the value system and the concerns of decision makers, constituting what MCDA-C calls primary elements of evaluation (PEEs). This approach also encouraged stakeholders to speak at meetings, expanding the understanding of decision makers [28, 36]. At this stage, 88 PEEs emerged [S1 Table].

Once a significant number of PEEs were generated, and when the decision maker started repeating the same lines, it was time to move forward in-depth. This occurred by transforming each PEE into a concept. A concept is an objective that expresses the preferred direction of the decision maker associated with each PEE. Each concept is made up of two components, the present pole, and the opposite psychological pole. The present pole represents the objective that the decision maker wishes to achieve and to give it a sense of action; the opposite psychological pole incorporates the performance that the decision maker wishes to avoid in relation to the objective [49, 50].

The concepts were determined in the same way for PEEs, asking the decision maker to talk about why the PEEs are important. To prevent the decision maker from diverting their reasoning, the facilitator kept the label of the problem in the decision maker’s sight. Table 1 presents the first five PEEs identified and their respective concepts, where the reticence (‘…’) should be read as “preferred to” or “instead of.”

**Table 1:** Primary elements of evaluation and concepts.

PEE	CONCEPT		
	PRESENT POLE	**…**	OPPOSITE PSYCHOLOGICAL POLE
Occupational Safety Policy	Promote an effective occupational safety prevention culture at all levels of the company	**…**	Ignore and neglect aspects of Safety at Work, increasing the number of accidents at work
Definition of Occupational Safety Values	Acting in a committed manner in our beliefs and valuing safe working conditions	**…**	Act insecurely, disregarding beliefs, not valuing safe working conditions
Management Engagement with Safety at Work	Level the values with the Management regarding the Occupational Safety Policy	**…**	Generate uncertainties with Management regarding the Occupational Safety Policy
Management Engagement with Safety at Work	Level the values with the Operational Leadership in respect of the Occupational Safety Policy	**…**	Generate uncertainties with the Operational Leadership regarding the Occupational Safety Policy
Engaging Operational Leaders with Occupational Safety	Level the values with the operational employees regarding the Occupational Safety Policy	**…**	Generate uncertainties with operational employees regarding the Occupational Safety Policy

Based on the understanding generated by the contextualization, the facilitator together with the decision maker identified the Areas of Concern that bring together all the concerns and motivations present in the decision-making context in a strategy of “top-down” action. Areas of Concern can be understood as the strategic objectives in the context of Fundamental Points of View (FPVs), which represent a set of properties and characteristics of the context that the decision maker associates with one or more of his values, and considers each of these properties and characteristics as essential to management [28, 35, 36, 47].

**Fig 3:**
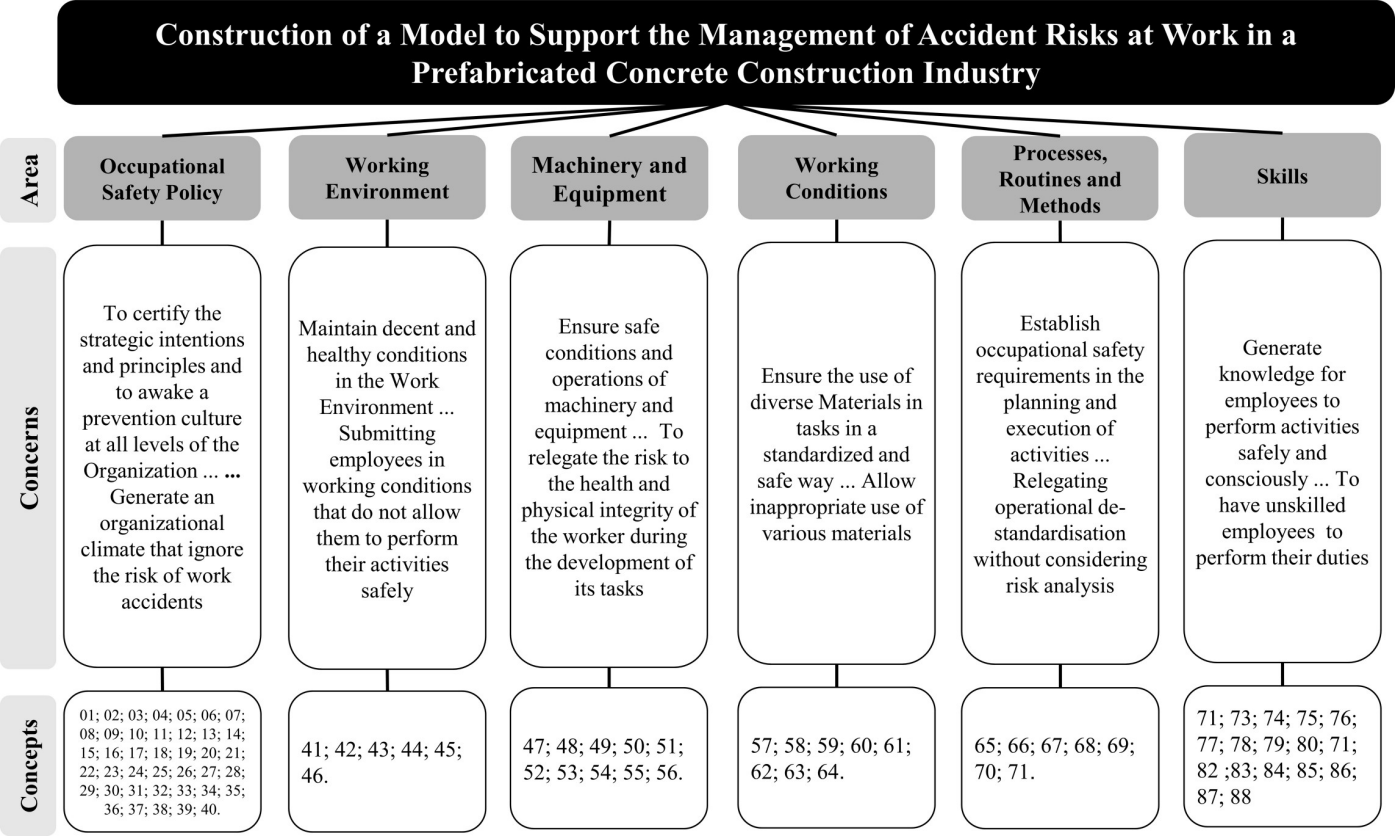
Grouping of concepts to form the FPV candidate family.

At this stage, six areas of concern were identified: (i) workplace safety policies, (ii) work environment, (iii) machinery and equipment, (iv) material conditions for work, (v) processes, routes, and methods, and (vi) skills. Once the adjustments were passed, these Areas of Concern passed the necessity (essentiality) and sufficiency (completeness) test. The value tree obtained at the end of the process is shown in Fig 3.

The area of concern, “Work Safety Policy,” addresses the engagement of the organization’s top management regarding aspects of work safety, prioritizing values, the form of dissemination to those involved, in addition to the strategies adopted to maintain the management’s commitment. Similar results where they consider the concern with the performance of work safety in organizations from the commitment of management were found in other studies [3, 14, 18, 51–54].

As for security management practices, the security training aspect pointed out by Vinodkumar and Bhasi [54] is also a strategic objective according to the decision-maker’s judgment. This concern was evidenced in the model through the “Skills” area of concern. In the model built, the decision-maker aims to improve the training of employees, focusing on the operational skills necessary for the development of activities, in addition to the qualifications required according to official regulatory standards. This same concern is evidenced in the studies by Fung and Tam [18], Hinze et al. [14], and Yiu et al. [16]. These authors warn of the importance of improving the workers’ skills through awareness of safety risks.

The decision-maker expressed concern during the development of the model about the importance of standardizing activities to ensure the reduction of work accidents. This aspect is evidenced by the area of concern “Processes, Routines and Methods.” This same concern is found in the research by Morgado et al. [53] in construction industries. In line with the model developed in this research, Fung et al. [3] warn about the practice of addressing safety rules and procedures. Fung and Tam [18], in their studies, report that effective safety instructions must be provided to workers. The same theme has been highlighted in other studies [14, 54, 55].

In addition, another aspect considered by the decision-maker in this area is the adequate inspection process, and inspections carried out by professionals in the occupational safety area to monitor the standardization of processes. Regarding inspection and safety inspections, the topic is addressed in the studies by Ilbahar et al. [22], Fernández-Muñiz et al. [55], and Li et al. [two].

Regarding the “Work Environment” area of concern, the decision-maker showed concern to ensure the mitigation of work accidents through care in the physical arrangement and access to the workplace, in addition to being concerned with keeping the environment clean and organized, and finally, alerting to ergonomic issues at work. Fernández-Muñiz et al. [55] and Yiu et al. [16] warn about the importance of these factors in the management of safety at work.

Machine protection systems, blocking systems, and the adequate operational functionality of the equipment are portrayed in the area of concern “Machines and Equipment.” The “Conditions of Materials for Work” area of concern demonstrates the decision maker’s concern to ensure the safety of workers with the correct use of accessories in industrial processes, in addition to the correct use of personal protective equipment by workers. Morgado et al. [53] identify that the lack of use of personal protective equipment by workers compromises the benefits of the occupational safety management system.

These areas of concern represented the aspects that the decision maker considered strategically essential (necessary and sufficient) to evaluate actions in the context. Thus, using the nomenclature proposed by Bana e Costa in 1992 [56], these were the candidates to form a family of fundamental points of view (FFPV) constituting the properties of consensuality, intelligibility, cohesion, completeness, monotonicity, and non-redundancy [47]. Together with the decision maker, the facilitator verified the compliance of these properties, and concluded that these areas of concern are suitable to be considered FFPVs. They were, therefore, capable of being evaluated by an Additive Aggregation model, more specifically by a Multicriteria Aggregation to a Single Synthesis Criterion model [27].

#### 3.1.3. Means-end maps and tree of fundamental viewpoints

To operate the FPVs, one of the tools used to build visibility of the context was the Cognitive Map (MC). It is an instrument for reflection, expansion of knowledge, and context analysis to organize and develop the decision maker’s understanding of the problem [27, 42, 49]. Cognitive maps hierarchically organize the concepts in a means-ends relationship [27, 47].

**Fig 4:**
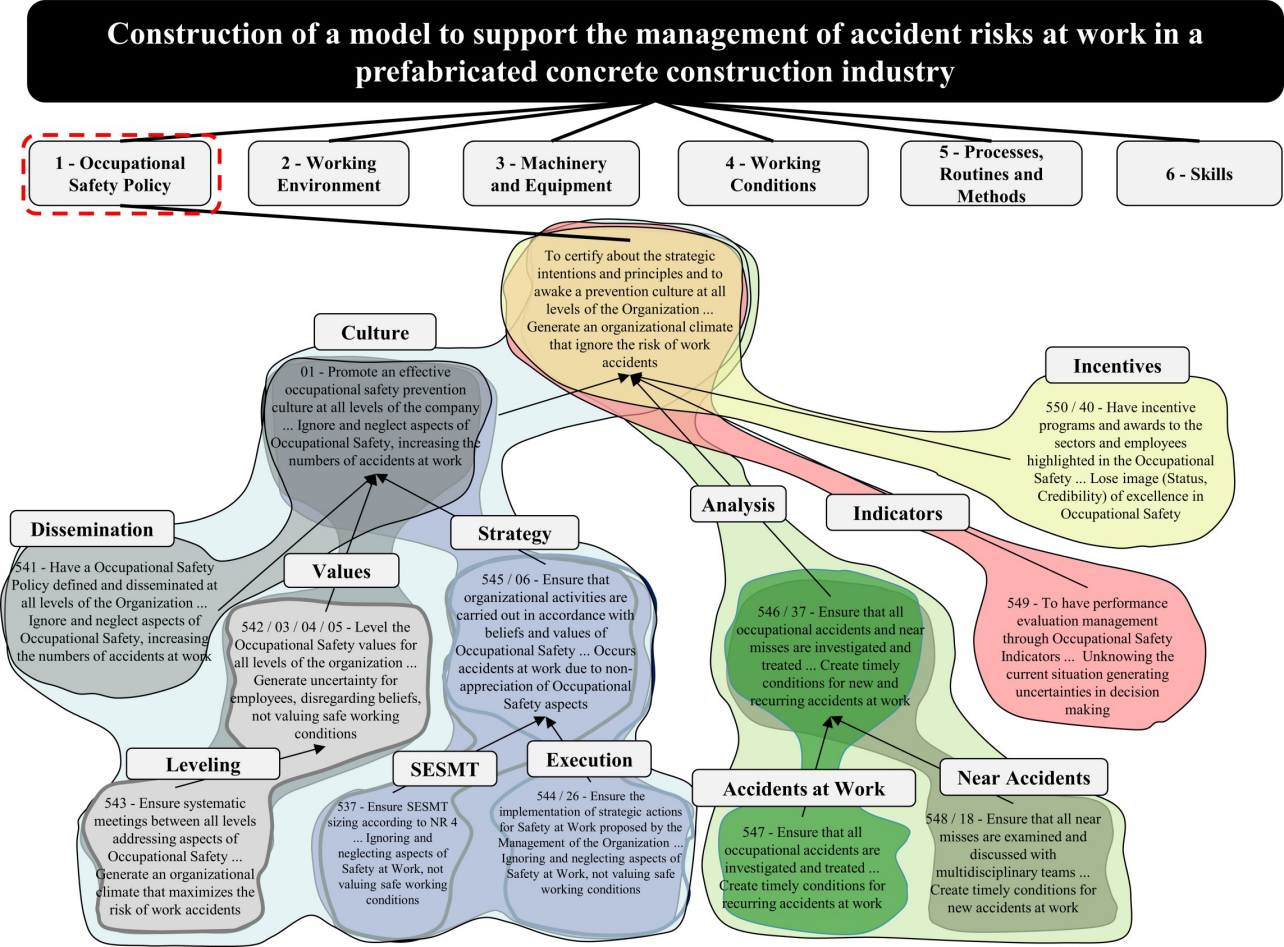
Clusters and subclusters of the Cognitive Map of the FPV “Work Safety Policy”.

In developing this process, the decision maker was encouraged to assess whether the structure under construction represents their understanding of the context. The MC concepts of each FPV were grouped by compatibility of concepts in clusters and subclusters for facilitating their understanding. These clusters were identified in such a way that the names represented the concern of the decision maker when developing the set of concepts associated with it, as portrayed in Fig 4.

The PVF Cognitive Map—Occupational Safety Policy contains aspects related to the culture of safety at work, careful analysis and investigation of work accidents, performance evaluation management, and promoting incentive programs with safety awards.

The “culture” cluster, which aims to promote a preventive culture of work safety at all organizational levels, considers the need to disseminate information in an agile and efficient way, in addition to guaranteeing the leveling of this knowledge and ensuring the execution of the strategic actions defined by the top management of the organization. In line with the results, Hinze et al. [14] conclude that security performance increased as the number of security practices increased. Regarding practices, the involvement of management in policy formulation was evident. Fernández-Muñiz et al. [52] study the role of safety leadership and proactive risk management in improving safety performance at work.

Ensuring that all work accidents or near misses are investigated to prevent recurrence is the concern of the decision-maker in the “analysis” cluster. This consideration was evidenced in the research by Li and Guldenmund [57]. It states that the safety management system is mainly driven by the analysis and prevention of accidents. Chi and Han [58] incorporate systems theory into Heinrich’s “Domino Theory” [15] model to better analyze the relationships between construction safety risks and reveal the causal chain of accidents. Thus, safety managers can prioritize risk factors according to the probability of the occurrence of accidents and the characteristics of injuries to control significant risks to achieve a safer work environment [58].

The “indicators” cluster demonstrates the decision-maker’s attention to having a performance evaluation management using safety indicators. This topic was the scope of work by Hinze et al. [59]. In Levenson’s research [19], he proposes an approach to identify and monitor safety indicators and provide guidance in designing a risk management framework. The application of a method based on prioritization and selection of the main indicators that measure the operational performance of work safety is the north of Podgórski’s studies [20]. Guo et al. [21] develop a theoretical model that conceptualizes the level of safety and facilitates the design of key indicators in the construction industry.

Seeking programs for incentives and awards for sectors and outstanding workers in occupational safety, the “incentives” cluster represents the decision-maker’s concern. This result is in line with the findings of Vinodkumar and Bhasi [54] when proposing the construction of a safety system model. It is considered one of the essential practices for the incentives and promotion of safety at work.

Each cluster, represented a concern of the context, and addressed the properties of being essential, controllable, complete, measurable, operational, isolated, non-redundant, concise, and understandable [25, 28, 35, 36, 38].

#### 3.1.4. Hierarchical value structure and descriptors

The set of concepts that formed a cluster defined and explained an area of interest related to the problem. Thus, a cognitive map of means-end relationships with numerous clusters could be simplified to a hierarchical value structure (HVS) [36], which also made it possible to portray the understanding of the decision maker’s value judgments in the model [38]. At HVS, the clusters, according to the MCDA-C methodology, were called elementary points of view (EPVs) [35].

With the hierarchical value structure built, each elementary point of view at the base of the HVS was perceived to be associated with a cluster that represented a tangible property. Therefore, it was capable of identifying the scale that measures its performance. The understanding generated by the cluster that gave rise to the EPV should support the construction of the ordinal scale that will be the descriptor or the performance indicator [50]. The descriptors (scales) can be qualitative, graphic, pictorial, or even represented by alphanumeric symbols. However, they need to meet the principles of objectivity, accuracy, and precision [25, 36, 47].

The model built for this study had six FPVs, consisting of 58 descriptors. To build the scales, the decision maker was asked to discuss the clusters, subclusters, and their concepts, and to present what would be excellent, good, normal, bad, and minimum acceptable performance. From this discussion, the facilitator suggested scales that, once approved by the decision maker, were considered descriptors. Once the scale represented their understanding of what they thought was important to measure, the decision maker was asked to establish the reference levels: the so-called "Good" and "Neutral" thresholds. These levels allowed for greater intelligibility of the decision maker’s absolute preferential value judgment [35, 47].

With the hierarchical value structure complete and operationalized, the performance profile for each of the scales was identified. The performance profile represented the current situation (status quo) of the context and the goals, i.e., the performance desired by the decision maker for each property associated with the descriptor.

When the performance evaluation is used to support the decision via the construction of an aggregation model to a single synthesis criterion, the use of measurement scales (that meet the Foundations of Measurement Theory in its empirical and theoretical aspects) is required. These requirements are necessary to ensure that the information generated from the model is not distorted [25, 60].

In the final stage of the structuring phase, the 58 descriptors were tested in terms of their faithfulness to the Measurement Theory Foundations, in line with the Empirical and Formal Mathematical Foundations [25, 61]. To respond to the empirical fundamentals, the scales were verified to ensure objectivity, precision, and accuracy (legitimacy), and were operationalized through the following properties: (i) unambiguity; (ii) intelligibility; (iii) operationality; (iv) measurability; (v) homogeneity; and (vi) availability of information (at each level) that allowed us to identify what is needed to reach the next level [27, 38].

In the structuring phase, the MCDA-C allowed the performance to be measured in an ordinal and isolated way from the values judged by the decision maker as essential (necessary and sufficient). In continuity with the knowledge construction process, in the evaluation phase, the model measured the performance of the context cardinally. This required incorporating information on the difference in attractiveness between the levels of the descriptors, transforming them into criteria (interval scales), and integrating them by means of compensation rates.

### 3.2. Evaluation

The assessment phase aimed to build a quantitative multidimensional model, where each FPV was weighted according to its contribution to assessing the overall performance of the model under study [18]. This mathematical model was a result of the following steps: (i) independence analysis, (ii) construction of value functions, (iii) identification of compensation rates, (iv) identification of the impact profile of the alternatives, and (v) sensitivity analysis [35, 42, 49, 50].

#### 3.2.1. Preferential independence test

The MCDA-C uses the aggregation method to a single synthesis criterion for performance measurement purposes. Thus, for the method to have scientific validity, its rates and scales must meet the mathematical requirements, and the criteria must preferably be independent, that is, isolable [25, 28]. Isolability, requires testing all scales to ensure that the attractiveness of moving from the lower to the upper reference level remains constant regardless of performance on the other scales [36].

Tests were performed for all peer-to-peer criteria, concluding that they are mutually independent (ordinarily and cardinally), and ensuring that compensation rates are constant for pre-established reference levels. In this way, the construction of an aggregation model to a single synthesis criterion for research has a legitimate application.

#### 3.2.2. Value functions

At the end of the preferential independence tests, the construction stage of the value functions began. Its objective was to transform the ordinal scales into cardinal scales that have the properties to conduct the mathematical operations required by the aggregation to a single synthesis criterion model. The transformation of ordinal scales into cardinal scales was achieved by incorporating information on the difference in attractiveness between all pairs of descriptor levels [35, 47]. For the decision maker, a value function had the meaning of numerically representing the level of attractiveness of a share; that is, the value function represented a value judgment and assisted the articulation of the decision maker’s preferences. This allowed the evaluation of potential actions according to a certain point of view [36].

In this study, the method of judgment for differences in attractiveness was used to obtain the cardinal scales and was operated through the MACBETH software (Measuring Attractiveness by a Categorical Based Evaluation Technique) proposed by Bana e Costa and Vansnick [62]. MACBETH tests the consistency of the judgments expressed and detects sources of inconsistency when applicable, thereby facilitating the review of the judgments in question. It also proposes a numerical scale compatible with the absolute judgments of the evaluator [62], supporting the desire of the decision maker to build a cardinal scale according to their preferential judgment for the decision-making context.

**Fig 5:**
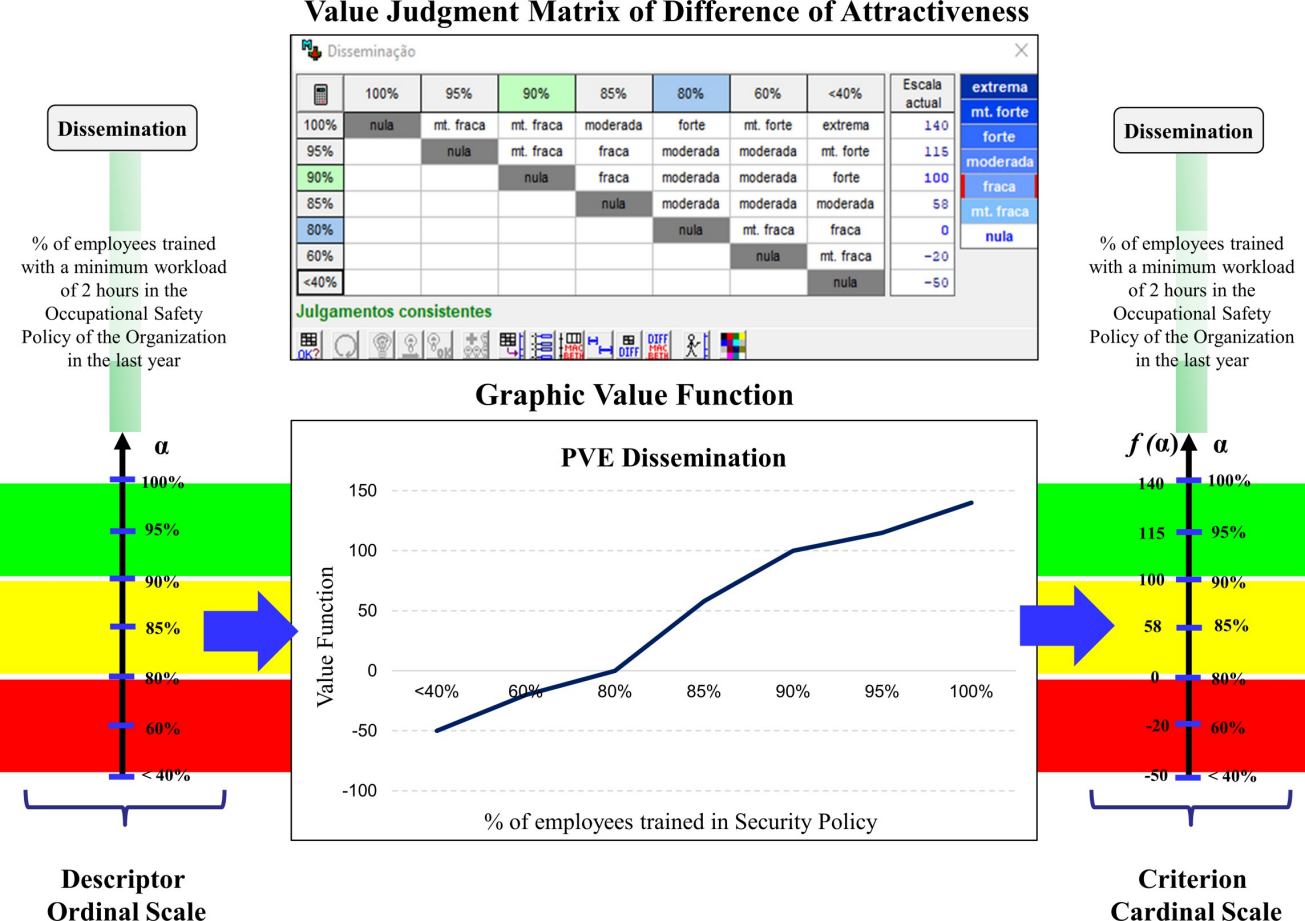
Transformation of the ordinal scale into the cardinal EPV “Dissemination”.

Fig 5 demonstrates the process of constructing the cardinal scale (value function) for the EPV descriptor “Dissemination,” and its transformation into a criterion, with emphasis on the attractiveness difference matrix (semantic judgment matrix). To fill in the data for the matrix, the decision maker used an ordinal scale proposed by MACBETH. The scale included the levels Extreme, Very Strong, Strong, Moderate, Weak, and Very Weak to express the difference in the attractiveness of performance going from a lower to a higher level among all combinations of the levels of the scale. The MACBETH software then presented a scale that met all the required proposed judgments in the matrix. This scale is not unique, and the facilitator can propose adjustments that the software itself will guide as to its feasibility.

When the construction phase of the value functions for all the descriptors of the model was completed, the criterion of this new function was renamed. Thus, local evaluation of all possible actions in the context was made plausible. However, for global evaluation, it was necessary to integrate these criteria, which was conducted according to the MCDA-C protocol, via compensation rates.

#### 3.2.3. Compensation rates

The construction of compensation rates (also called substitution rates) aimed to inform the contribution of each criterion when the performance of an alternative passes from the “Neutral” level to the “Good” level. Based on the compensation rates of each criterion, an equation could be constructed that allowed an overall assessment of the evaluated context [30]. This way of understanding rates made its interpretation a scaling factor that turns local units into global units. The MCDA-C methodology proposes the following procedures for determining the rates: (i) highlighting of alternatives; (ii) ranking of alternatives; and (iii) determination of rates [27, 28, 35].

**Fig 6:**
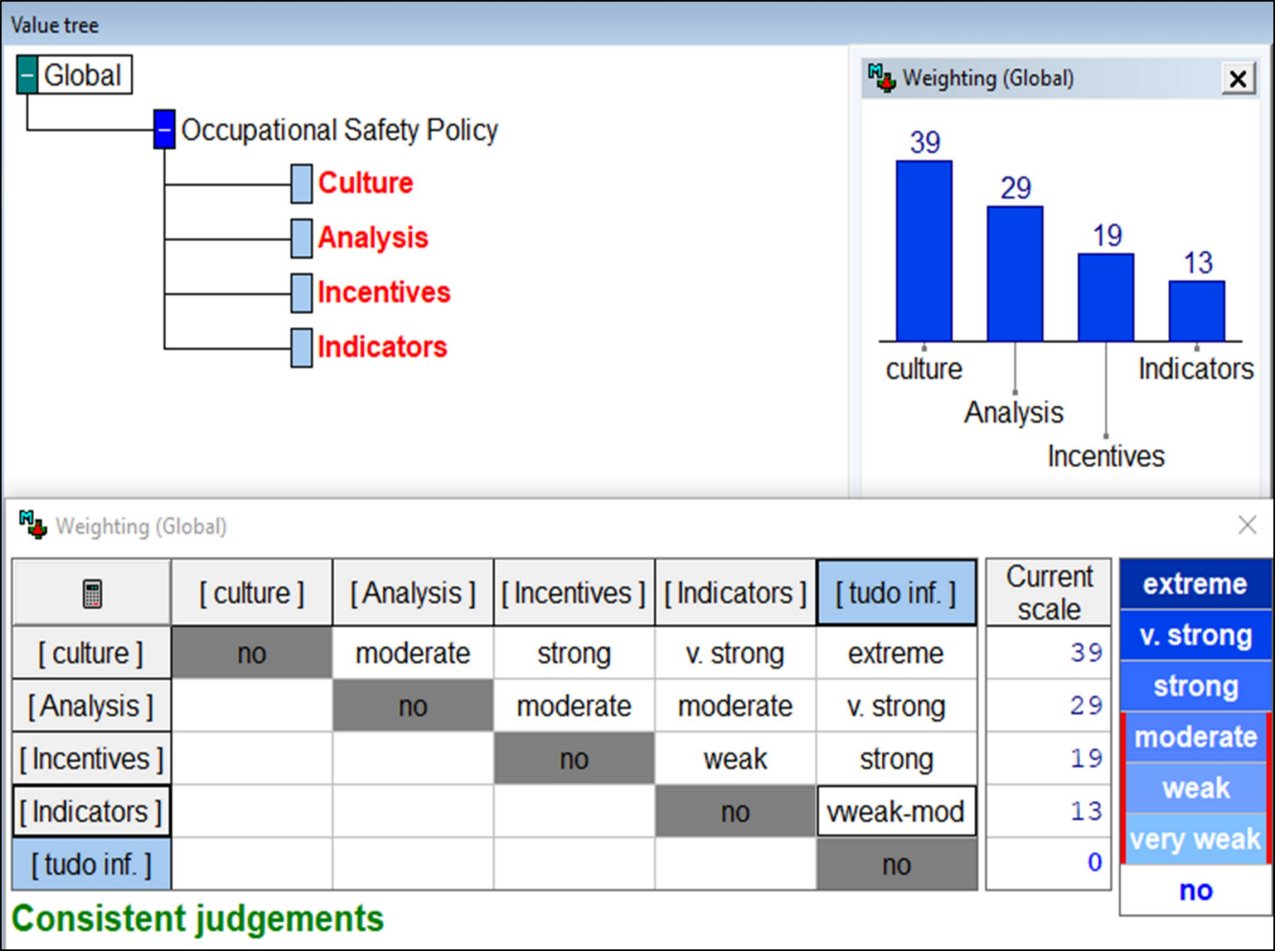
Determination of rates for the FPV “Occupational Safety Policy”.

With this understanding, the first step in determining compensation rates was to create fictitious shares with “Good” performance in one criterion and “Neutral” in the others. The Roberts Matrix was then used to support the decision maker’s ordering of the preference judgment. In this matrix, one could compare pairwise alternatives and assign value 1 for the preferred stock and value 0 for the other stock. After all the comparisons had been made, the values of the lines were added together and, the ordering of preference was obtained from the decision maker [35]. MACBETH was used to determine the compensation rates. The use of the semantic matrix of the difference in attractiveness between the alternatives numerically represented the semantic judgments made by the decision maker, as illustrated in Fig 6.

Thus, the hierarchical value structure (HVS) of the FPV “Occupational Safety Policy” had the appropriate rates determined. With this information, one established the performance equation for the FPV “Occupational Safety Policy” where:

$$V_{Polícy}{\left(a\right)}_{}=0,39*V_{Culture}(a)+0,29*V_{Analysis}(a)+0,13*V_{Indicators}(a)+0,19*V_{Incentives}\left(\text{a}\right)$$

Looking at the formula, it is possible to identify the decision-maker’s preferences regarding each area of concern. The model indicates that for the reference levels considered, the area that most contributes to the performance of the PVF “Work Safety Policy” is the “Culture” area corresponding to 39% of the overall performance, followed by “Analysis,” with 29% and “Incentive” with 19%. The “Indicators” area of concern was less representative of its impact on security policy performance (13%).

#### 3.2.4. Global assessment and current situation profile

**Fig 7:**
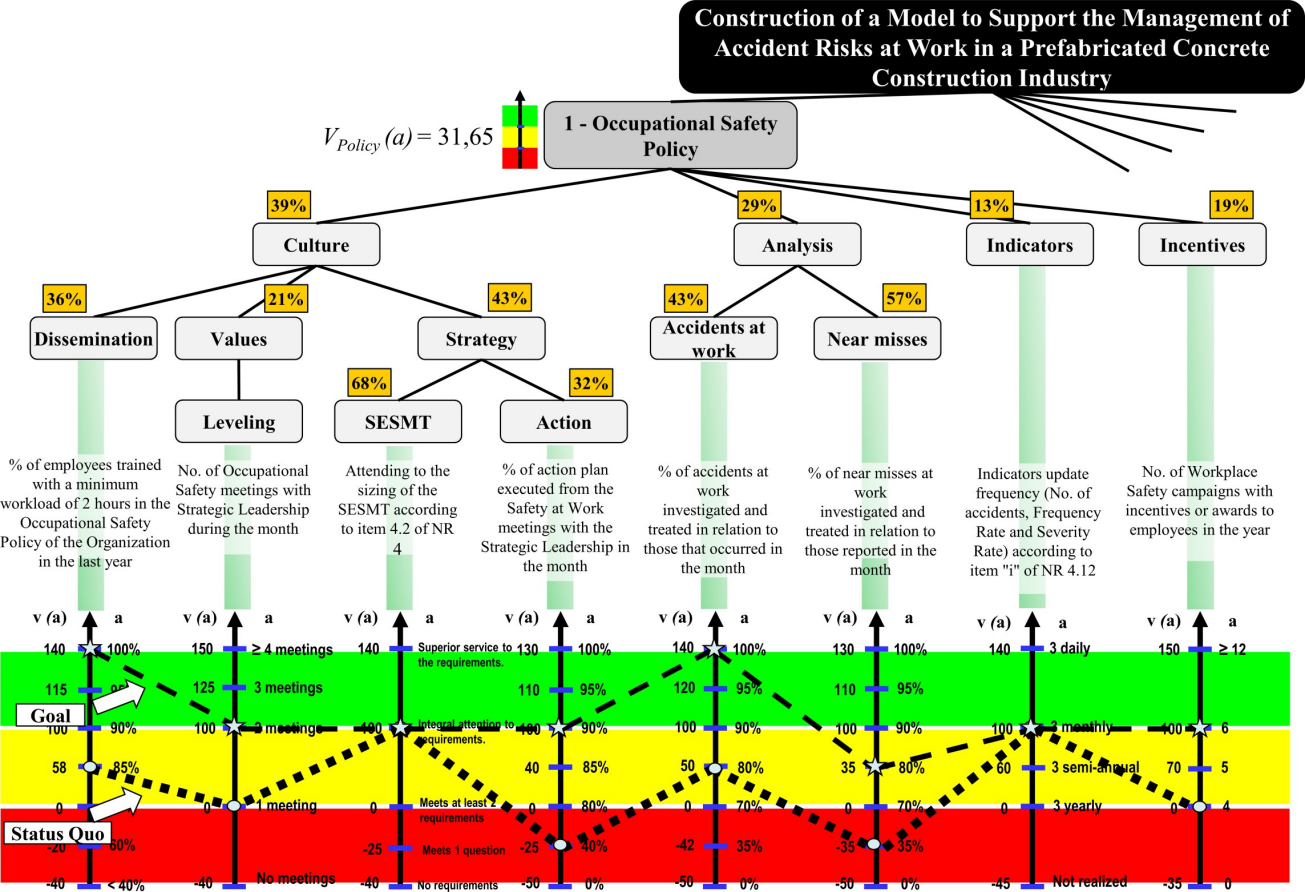
HVS “Occupational Safety Policy” with status quo display.

Once the HVS was built and the compensation rates associated with the criteria were determined, this knowledge was used to evaluate the status quo impact profile globally. The overall performance of the model is calculated from the additive aggregation formula by adding the partial performance values of each criterion to the alternative (a), weighted by substitution rates [35]. For the Global Assessment of the FPV “Occupational Safety Policy,” it was necessary to determine the local performance of each criterion from the status quo profile, according to Fig 7.

By determining the status quo of each criterion, it was possible to obtain an overview of the strengths and vulnerabilities of the model criteria, thus allowing monitoring of the performance of SQ in aspects deemed essential by the decision maker. To conduct the overall appraisal for the FPV “Occupational Safety Policy,” the equation generated in the previous section was used to replace the V(a) values determined for each EPV with the impact profile value by the status quo appraisal, that is:

$$V_{Policy}{\left(a\right)}_{}=\left\{0,39*\left[0,36*V_{Dissemination}(a)+0,21*V_{Leveling}(a)+0,43*\left(0,68*V_{SESMT}(a)+0,32*V_{Action}(a)\right)\right]\right\}+\left[0,29*\left(0,43*V_{Accident}(a)+0,57*Q_{near-accident}(a)\right)\right]+\left(0,13*V_{Indicators}(a)\right)+\left(0,19*V_{Incentives}(a)\right)$$

$$V_{Policy}{\left(a\right)}_{}=\left\{0,39*\;\left[0,36*58+0,21*0+0,43*\left(0,68*100+0,32*\left(-25\right)\right)\right]\right\}+\left[0,29*\left(0,43*50+0,57*\left(-35\right)\right)\right]+\left(0,13*100\right)+\left(0,19*0\right)$$

$$V_{Policy}{\left(a\right)}_{}=18,20+0,45+13+0$$

$$V_{Policy}\left(a\right)=31,65$$

The value of the score had as reference to the value 0 to represent the performance boundary between Normality and Commitment. The reference to the value 100 represented the threshold between Excellence and Normality performances.

### 3.3. Recommendations

The recommendation phase aimed to support the decision maker in identifying the best opportunities to improve performance. Thus, the first activity in this phase consisted of graphically and numerically visualizing the criteria with the performances at the compromising level, and calculating the contribution (score) of passing from this level to the desired goal [27, 28, 50]. Once the criterion that provided the greatest contribution was identified, the facilitator identified the property that has its performance measured by the criterion and requested the decision maker’s help in identifying actions to move this property from current performance to target performance [35].

**Fig 8:**
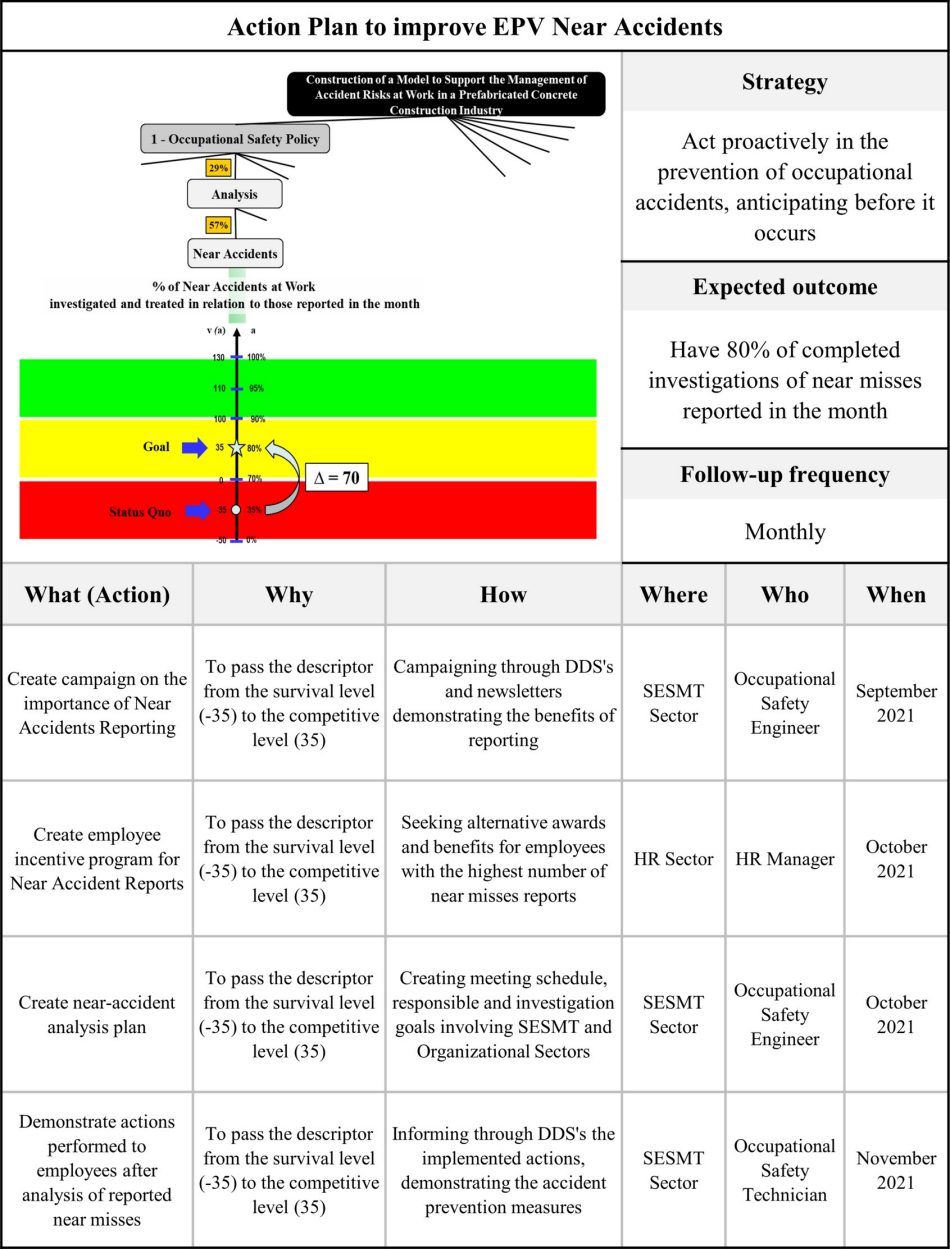
Action plan to raise the performance of criterion investigation and treatment of near accidents.

The action plan developed is an adaptation of the 5W2H quality tool (what, when, who, where, why, how, how much). It is a simple method, adaptable according to each situation, and used to describe action plans carefully and objectively, thus ensuring their organized execution [63]. Fig 8 shows the improvement actions generated for the criterion of near-accidents (EPVs), measured by the decision maker as a percentage of near accidents at work investigated and treated compared to those reported in the last month.

This process was repeated for the other criteria, highlighting the potential contribution that the proposed model made available to the organization. The knowledge provided by MCDA-C allowed identifying the aspects judged by the decision maker as essential, measuring their performance, giving visibility to the performance of the current situation, setting the goal, supporting the decision-making process of managing occupational accident risks, and monitoring and generating actions to improve the performance of those properties whose performance did not meet the expectations of the decision maker. In this way, by possessing the proposed action plan, setting goals, and identifying criteria with a higher level of contribution, the decision maker had a tool that allowed them to carry out the planning and monitoring of improvement actions in a structured way.

## 4. Conclusions

Civil construction is an activity that has been associated with human beings since its emergence. The exponential population growth in the last five decades has triggered the need for housing and infrastructure in the same proportion. Unfortunately, this growth in the construction process has occurred without a parallel scientific development of workers’ safety. This has left an annual legacy of millions of people dead or with permanently disabling injuries accompanied by the respective social and economic repercussions. In this context, this research aimed to build a multicriteria model for decision making to support the management of occupational accident risks in the concrete prefabricated construction industry. The MCDA-C instrument was chosen for its ability to deal with complex problems that require structuring, evaluation, and recommendation to monitor and improve decision-making environments in a unique way.

In the structuring phase, the construction of the model allowed identifying (i) the decision-making environment, (ii) the actors involved in the process, (iii) the definition of the label, (iv) the determination of 88 PEEs, whose concepts were grouped into six areas of concern that represent the following strategic objectives of the organizational context: occupational safety policies; work environment; machinery and equipment; material conditions for work; processes, routes, and methods; and skills. All of these were covered in addition to building the hierarchy of concepts toward the means and ends, represented by FVPs.

In the evaluation phase, MCDA-C made possible the migration of a qualitative model made up of descriptors with ordinal scales to a model with cardinal scales. This process consisted of building value functions and determining the compensation rates that allowed them to be aggregated. Thus, the process provides the measurement of local performance as well as overall performance, operated via 58 criteria.

All this knowledge associated with the status quo profile identified gave visibility to the process of monitoring and identifying opportunities. With reference to the criteria for performance at a compromising level, the use of the information contained in the model allowed the development of actions for its improvement and measurement of its local contribution, finalizing the recommendations phase.

In terms of limitations of the present study, the model was constructed from the perception, values, and preferences of the decision maker for a specific decision-oriented environment. Therefore, the model needs to be adjusted as per other social contexts, particularly in terms of the physical environment and the values and preferences of the new decision maker.

In continuation with the knowledge construction process originated by the research, it is suggested, for future research, that a risk management model be evolved using the MCDA-C methodology for other economic sectors that share high rates of occupational hazards. This could apply especially well to the hospital care sector in Brazil, given its social contribution.

### 4.1. Practical and theoretical contributions

Developing an academic/scientific work to meet a practical demand is simultaneously a pedagogical process and a challenge to respond to the demands of the fragment of professionals who work with the theme, in the expectation that the results contemplate both demands. The present research contributes to both.

The academic/scientific community is able to contemplate/witness the construction of a management support model for a partially known, confused, and nebulous complex context, with specific singularities that need to be present in the model, such as: (i) there are multiple actors with conflicting interests in influencing its management; (ii) the decision-maker, like the other actors, does not have clearly established objectives but wants to expand his understanding of how the context affects his values, preferences, motivations and concerns; (iii) the decision-maker wants, during the process of building the model, to establish a debate forum so that everyone can be heard, even if the last word is his, since the consequences of the decisions will fall on him; and (iv) the decision-maker wants to be able to highlight the strategic objectives when managing safety and have scales to measure the performance of each intervening factor besides knowing the current situation/performance, establishing goals and, with legitimacy and scientific basis, build actions of improvement.

Since no publication with such a profile was found for the topic of risk management of accidents at work in the prefabricated concrete construction industry, it can be said that this research contributes to the academic/scientific community.

The community of practical users of decision support instruments, when seeking support to reinforce their decisions, has received generic models that do not represent the characteristics and singularities of their contexts and, therefore, their use frustrates decision makers/users [25]. The present research overcomes these limitations and provides decision-makers with a legitimate model of the manager’s concerns, motivations, and values. It considers the particularities of the environment and measuring the performance of the factors considered by the manager as essential, with scales that meet the scientific foundations of the Theory of Measurement. Furthermore, the present model, when revealing the performance of each critical property, provides an instrument to establish the goal and generate improvement actions.

In this way, the theoretical and practical contributions of the results of the construction of this model contribute to researchers/authors, the academic/scientific community, and society.

## Supporting information

S1 Table Primary elements of evaluation. (DOCX)
