# Supplementary material for: Constructivist multi-criteria model to support the management of occupational accident risks in civil construction industry
Source: PLoS One. 2022 Jun 28;17(6):e0270529. doi: 10.1371/journal.pone.0270529 (PMC9239452; doi:10.1371/journal.pone.0270529)
Supplement: S1 Table — (DOCX) [file pone.0270529.s001.docx]

| **Primary Elements of Evaluation - PEEs** | | | | | | | | |
| --- | --- | --- | --- | --- | --- | --- | --- | --- |
| **01** | Occupational Safety Policy | **02** | | Definition of Occupational Safety Values | **03** | Management Engagement with Safety at Work | **04** | Management Engagement with Safety at Work |
| **05** | Engaging Operational Leaders with Occupational Safety | **06** | | Employee engagement with Work Safety | **07** | Employee satisfaction and motivation with work | **08** | Employees' confidence in work activities and routines |
| **09** | Work pressure exerted by leadership | **10** | | Fatigue and tiredness during the working day | **11** | PPRA - Environmental Risk Prevention Programs | **12** | PCMSO - Medical Control and Occupational Health Program |
| **13** | SESMT Dimensioning - Specialized Service in Safety and Health at Work | | **14** | Qualification of SESMT professionals | **15** | Emergency Brigade | **16** | CIPA - Internal Commission for Accident Prevention |
| **17** | CIPA Sizing | | **18** | CIPA implementation process and activities | **19** | Actions carried out by CIPA | **20** | Absolute Number of ACA - Work Accidents With Leave |
| **21** | Absolute Number of ASA - Accidents at Work Without Leave | | **22** | Number of Near-Misses | **23** | Number of Unsafe Acts | **24** | Number of Unsafe Conditions |
| **25** | Frequency Rate of Accidents With Absence | | **26** | Frequency Rate of Accidents Without Leave | **27** | Accident Severity Rate | **28** | Number of VIS - Work Safety Surveys |
| **29** | Results of Compliant Items in VIS - Work Safety Surveys | | **30** | Work Safety Meetings | **31** | Work Safety Action Plan | **32** | EPIs - Personal Protective Equipment |
| **33** | Use and monitoring of EPIs - Personal Protective Equipment | | **34** | ASO - Occupational Health Attested | **35** | APR - Preliminary Risk Analysis | **36** | EN - Work Permit |
| **37** | PPRA Annual Planning - Environmental Risk Prevention Program | | **38** | Anticipation and recognition of risks | **39** | Quantitative assessment of the risks of exposure to workers | **40** | Qualitative assessment of the risks of exposure to workers |
| **41** | Control and evaluation measures | | **42** | Risk exposure monitoring | **43** | Registration and disclosure of data | **44** | Environmental Conditions |
| **45** | Physical Arrangement and Facilities | | **46** | Cleaning and Organization of the area | **47** | Means of access | **48** | Work Safety Signage |
| **49** | Ergonomic aspects | | **50** | Machines and Equipment in perfect conditions of use | **51** | Moving parts of Machinery and Equipment | **52** | Machinery and Equipment Safety Systems |
| **53** | Machinery and Equipment Emergency Stop Devices | | **54** | Machinery and Equipment Inspections | **55** | Machine and Equipment Maintenance | **56** | Machinery and Equipment Adjustments |
| **57** | Machinery and Equipment Lubrication | | **58** | Cleaning of Machine and Equipment | **59** | Machinery and Equipment Manuals | **60** | Material Conditions |
| **61** | Work tools | | **62** | Conditions of steel cables | **63** | Conditions of lifting lock systems | **64** | Conditions of lifting rockers |
| **65** | Conditions of shims and tweeours for storing parts | | **66** | Raw Material Conditions | **67** | Operational Procedures for Performing Tasks | **68** | Work Safety Procedures |
| **69** | Machinery and Equipment Operation Procedures | | **70** | Material Handling Procedure | **71** | Parts Storage Procedure | **72** | Emergency Response Plan |
| **73** | Integration Training | | **74** | Training of Operational Procedures | **75** | Training of Work Safety Procedures | **76** | TRAINING OF APRs - Preliminary Risk Analysis |
| **77** | DDS – Day security Dialogues | | **78** | Training NR 01 - Service Order | **79** | NR Training 06 - Use of EPIs | **80** | NR 10 Training - Electricity installations and services |
| **81** | NR 11 Training - Material Handling | | **82** | Training NR 11 - Crane Operation | **83** | NR 12 Training - Machinery and Equipment | **84** | Training NR 12 - Rotating machines |
| **85** | Training NR 18 - Working Environment Conditions in the Construction Industry | | **86** | Training NR 20 - Activities with Flammables and Fuels | **87** | Training NR 35 - Work at Height | **88** | Availability of Work Safety Training |
